# Asymmetric host movement reshapes local disease dynamics in metapopulations

**DOI:** 10.1038/s41598-022-12774-5

**Published:** 2022-06-07

**Authors:** Matthew Michalska-Smith, Kimberly VanderWaal, Meggan E. Craft

**Affiliations:** 1 Department of Veterinary Population Medicine, University of Minnesota, St. Paul, MN USA; 2 Department of Plant Pathology, University of Minnesota, St. Paul, MN USA; 3 Department of Ecology, Evolution, and Behavior, University of Minnesota, St. Paul, MN USA

**Keywords:** Computational models, Theoretical ecology, Ecological networks, Ecological modelling, Ecological epidemiology

## Abstract

Understanding how the movement of individuals affects disease dynamics is critical to accurately predicting and responding to the spread of disease in an increasingly interconnected world. In particular, it is not yet known how movement between patches affects local disease dynamics (e.g., whether pathogen prevalence remains steady or oscillates through time). Considering a set of small, archetypal metapopulations, we find three surprisingly simple patterns emerge in local disease dynamics following the introduction of movement between patches: (1) movement between identical patches with cyclical pathogen prevalence dampens oscillations in the destination while increasing synchrony between patches; (2) when patches differ from one another in the absence of movement, adding movement allows dynamics to propagate between patches, alternatively stabilizing or destabilizing dynamics in the destination based on the dynamics at the origin; and (3) it is easier for movement to induce cyclical dynamics than to induce a steady-state. Considering these archetypal networks (and the patterns they exemplify) as building blocks of larger, more realistically complex metapopulations provides an avenue for novel insights into the role of host movement on disease dynamics. Moreover, this work demonstrates a framework for future predictive modelling of disease spread in real populations.

## Introduction

Many populations of humans, livestock, and wildlife are comprised of densely occupied subpopulations, or “patches”, connected by the movement of individuals, i.e., a metapopulation. Most of the world’s population lives in cities^1^, livestock are clustered on farms^2^, and wildlife tend to cluster in space, especially when usable habitat is fragmented^3,4^. For humans, movement between patches was once at such a low level that emerging diseases tended to be geographically constrained to the patch where they originated^5^. For instance, the strain of *Yersinia pestis* that resulted in The Black Death, was constrained to Europe for nearly four centuries before being introduced in China^6^. Likewise, Smallpox remained relatively isolated in subpopulations around Europe until the Crusades of the 11th and 13th centuries made it endemic across the continent, and it took until the 16th century for the pathogen to tag along colonizing expeditions the Americas^7^. The world today, in contrast, is increasingly connected, allowing diseases like Ebola^8^, Influenza^9,10^, and COVID-19^11,12^ to spread more widely, and more quickly, than ever before.

Human movement has consequences beyond the spread of human-specific pathogens as well, being key to both the spread of wildlife-associated pathogens between otherwise isolated habitats^13,14^ and the spread of diseases between livestock populations via direct transport of infected animals^15^ or through contaminated vehicles or equipment^16^. Even in the absence of human-mediated spread, diseases in livestock and wildlife populations can have dramatic consequences on human populations through spillover, economic loss, and reduction of ecosystem services^14^. Thus, a better understanding of how population movement (human or otherwise) affects disease spread is critical to preventing and responding to future epidemics.

Host movement is critical both to the spread of disease across landscapes and to the persistence of pathogens in the populations they infect^17–22^. Initially introduced through the concept of island biogeography^23^, a network approach to modelling metapopulations lends itself readily to the study of empirical systems, such as human movement between cities^24^, livestock transport between farms^15,25^, or wildlife living in fragmented natural habitats^26^. Representing a metapopulation of cities, for example, involves mapping each city to a node in the network and connecting those nodes with edges when there is movement of individuals between them. A network-based metapopulation framework can facilitate characterization of the relationships between connected patch dynamics as well as characterize the structure of the system as a whole, providing unique insights across scales^27,28^.

Importantly, differences in local parameter values (such as carrying capacity or transmission rate) can lead to patches within a metapopulation exhibiting disparate dynamical regimes, e.g., one patch might exhibit steady pathogen prevalence through time, while another might oscillate between high and low prevalence. These differences between patches can be particularly important when cycling or chaotic dynamics result in temporarily low local population or pathogen densities. Timely influx of individuals from asynchronous patches at such moments has the potential to rescue patches from local extinction, or provide the boost needed to ensure pathogen persistence^26,29–33^. Moreover, understanding the dynamics of a particular patch (and its relationship to the rest of the metapopulation) is essential to developing appropriate interventions to limit further disease spread. For instance, if pathogen prevalence is cycling, timing an intervention during a lull in prevalence can improve both efficacy and cost efficiency^34^.

The study of metapopulations has a rich and expansive history in Ecology. In the field of consumer-resource dynamics, for instance, metapopulation models have revealed the importance of spatial heterogeneity for the stability and persistence of complex ecological communities, e.g., by providing prey refugia or generating an implicit density-dependence in population growth rates^35,36^. Likewise, movement between heterogeneous patches can allow for the exchange of individuals between asynchronously varying populations, stabilizing both consumer-resource^35,36^ and disease dynamics^37,38^. Such mechanisms have been considered as potential answers to the naturally destabilizing forces of stochasticity and time-lags that are intrinsic to empirical systems, though comprehensive analytics of such systems has been lacking^39^.

In disease ecology and epidemiology, metapopulations have long been used to understand the spread of disease in complex population structures^40–42^. Most commonly, models have focused on connecting patches through pathogen transmission, rather than by the explicit movement of hosts between patches^2,43,44^, but see^27,28,45,46^. While more mathematically tractable, this abstraction omits the effects of population shifts (i.e., changes in local demographics and population sizes resulting from the movement of individuals between patches) on the disease dynamics, including, importantly, the movement of immune individuals. Furthermore, much of the focus thus far has been on persistence, and the positive effects of movement thereupon^35–38,47^. In contrast, categorizing the qualitative local dynamical regimes in a two-patch metapopulation of Ricker-modelled populations, excellent work by Dey et al.^48^ notes that host movement can be either stabilizing or destabilizing based on the dynamics of the origin patch. In this work, we build upon prior results, focusing on the categorical disease dynamics being experienced by a given patch in the presence or absence of host movement in both small and large metapopulations. Put simply, if a disease is exhibiting oscillatory dynamics in patch *A* and steady-state dynamics in patch *B*, does movement of individuals from *A* to *B* alter dynamics in *B*? Is the effect qualitatively different if the direction of the movement were reversed?

Using a simple disease model that can intrinsically (i.e., without environmental forcing) exhibit dynamics ranging from pathogen extinction (i.e., a disease-free equilibrium), to constant prevalence through time, to cycling or chaotic fluctuations in prevalence, we characterize the effects of host movement on disease dynamics in two parts. First, we consider the effect of simulating a continuous, proportional flow of inter-patch host movement (*sensu*^45^), along with disease dynamics, on artificially simplistic sub-networks constructed to exemplify key relationships between patches. These small, archetypal networks moreover serve as building blocks of larger, more complex metapopulations. We parameterize the patches in these networks to display either (A) identical or (B) disparate dynamics in the absence of host movement, and specifically look at how those dynamics might change when host movement is occurring. Finally, we consider the effect of host movement on larger, more complex metapopulation structures, in particular asking whether and how any patterns observed at small scales might affect dynamics when embedded in larger movement networks. The robustness of each result is examined by comparing multiple parameterizations of two underlying models of within-patch disease dynamics: a Susceptible-Exposed-Infectious (SEI) model^49^, and a compartmental model of multi-strain disease^50^. As results are qualitatively similar between model formulations, we present just one parameterization of the SEI model here and provide additional results in the Supporting Information.

## Results and discussion

### Archetypal sub-networks

**Figure 1:**
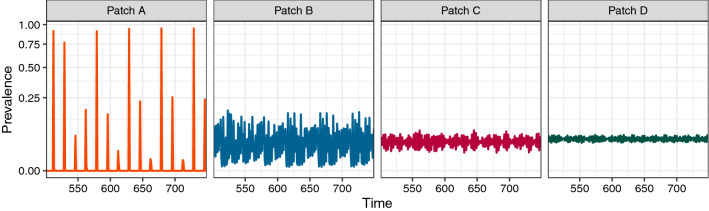
Connecting multiple patches with the same parameters and initial conditions results in reduced peak pathogen prevalence and dampened oscillations in patches further down the chain. Here, patches are connected such that $A\rightarrow B\rightarrow C\rightarrow D$. Each panel indicates the prevalence (i.e., the proportion of the patch population currently infected with the pathogen) over time in that particular patch. Because all patches have the same parameters and initial conditions (see Methods), all patches would have the same dynamics (i.e., cycles, as seen in^49^) in the absence of movement between patches. Thus, all differences between patch time series are due to immigration from and emigration to other patches in the chain. Note also that completing the circle (such that $A\rightarrow B\rightarrow C\rightarrow D\rightarrow A$) would again make all patches identical, removing any distinction between origin and destination patches. Transient dynamics are omitted from the time series for clarity.

First, we consider the case of a chain of interconnected patches which have been parameterized to display identical dynamics in the absence of host movement (i.e., individuals move from patch *A* to *B* to *C* to *D*, and all patches share the same disease parameters and initial conditions). We found that oscillations are dampened in subsequent patches relative to those in patch *A*, reducing peak pathogen prevalence (i.e., the proportion of the patch population currently infected with the pathogen; Fig. 1). This echoes results in ecological movement networks, where local population dynamics were dampened following the introduction of host movement^47,51^, as well as work in food-webs looking at weakly coupled predator and prey oscillators^52^. As the chain of patches is lengthened, however, the change in oscillation amplitude between subsequent patches did not necessarily continue to shrink with the addition of more patches. Concurrently, we saw an increase in the correlation between neighboring patches’ prevalences, the extent of which depends on the movement rate^53^ (Supporting Information Fig. S1).

When considered in the context of larger network structures, this dampening of oscillations suggests that prevalence in “source” patches (i.e., patches with only emigration) will tend to have greater variability as well as greater asynchrony with neighboring patches. Taken together, these pose an interesting tradeoff: at the patch level, the reduction in oscillation amplitude is generally viewed as stabilizing^49^, thus increasing the likelihood of pathogen endemicism. At the same time, the increase in synchrony between patches can be destabilizing at the metapopulation level, increasing the risk of pathogen extinction in low-prevalence patches when neighboring patch prevalence is also low^54–56^.

**Figure 2:**
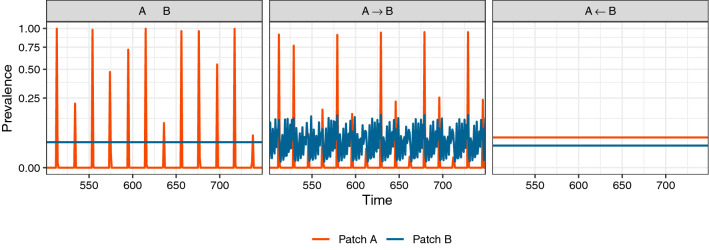
Destination patches tend to inherit origin patch dynamics when linking patches with different model parameterizations. Panels correspond to network structure, with line color indicating the prevalence (i.e., the proportion of the patch population currently infected with the pathogen) through time in particular patches. While in isolation (left column), patch *A* has oscillatory dynamics and patch *B* has steady-state dynamics (see Methods), when the two patches are linked by movement, the destination patch inherits the dynamics of the origin patch (center and right panels). This is true regardless of the direction of the movement (but does depend on the rate of movement; see Supporting Information Fig. S4). Transient dynamics are omitted from the time series for clarity.

Next, we consider a case in which patches are not equivalent (i.e., parameters differ between patches such that they exhibit distinct dynamical regimes in the absence of movement). When patches with disparate parameters (and thus dynamical regimes) are linked, the dynamics of destination patches can be overridden by the dynamics of origin patches (Fig. 2). While the introduction of oscillations to a steady-state patch might be expected, surprisingly, we found the opposite to also be true (i.e., steady-state dynamics overruling oscillations), though this requires a higher rate of movement (Supporting Information Fig. S4). Indeed, the cessation of oscillatory behavior, also termed “amplitude death,” has been observed even in the case of linking two entities experiencing cycling dynamics to one another^57–60^, though most of these studies consider the effect on the system as a whole (the entire metapopulation, in our case), rather than the effects on individual entities/patches.

**Figure 3:**
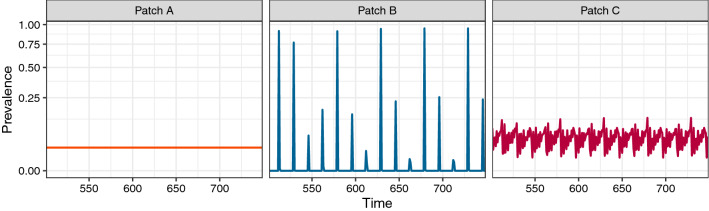
When multiple origin patches differ in their dynamics, the destination patch inherits oscillations over steady-states. As in Fig. 1, panels correspond to individual patches, with lines indicating the prevalence (i.e., the proportion of the patch population currently infected with the pathogen) through time. Here, we have patches *A* and *B* feeding into patch *C* at the same rate; $A \rightarrow C \leftarrow B$. *A* and *C* are parametrized to produce steady-state dynamics in the absence of movement (see Methods). *B* shows oscillatory dynamics, with all other parameters the same. Note that, even though the parameters of *C* would lead to a steady-state in the absence of movement, we see oscillatory dynamics being inherited from *B*. Transient dynamics are omitted from the time series for clarity.

Because we saw both oscillatory and steady-state dynamics propagating through the metapopulation, it is natural to ask what destination dynamics look like when there are multiple, varied origin patches for a single destination patch. In such cases, we observed a hierarchy of dynamics in regard to their propagation through the network: when there are origin patches with both oscillatory and steady-state dynamics, the destination patch inherited the oscillatory dynamics, albeit dampened from what they would have been without movement from a steady-state patch (Fig. 3). This asymmetrical inheritance was robust to imbalance in the relative contributions of the origins (Supporting Information Fig. S5).

The inheritance of dynamical regimes combined with a hierarchy that favors oscillatory dynamics suggests that these more volatile dynamics should be more common, especially in patches further “down the chain.” That is, except in cases where all source patches are disposed toward steady-states, in which case the stabilizing effect has the potential to overrule downstream local parameterizations, leading to an overall stable system. These results are consistent with, and provide a mechanism for, the longstanding observation that non-steady-state dynamics predominate in empirical disease systems^61,62^, and in population dynamics more generally^63^.

### Larger network structure

Empirical networks are much larger and more connected than these simple examples—are there consequences of these local patterns on the dynamics of larger, more complex metapopulations? Rather than restricting our analysis to a small sample of empirical movement networks (few of which contain directionality or rates of movement), we evaluated the effect of various global network structures through the use of five well-studied network ensembles. Depending on the system being explored, a given empirical network might have elements in common with one or more classical network structure ensembles, for instance, many social networks are considered to be “small-world” in structure like Watts-Strogatz random networks^64–66^, while ecological networks are often noted for their formation of “modules” or clusters of more densely interacting species as in stochastic block random networks^67–71^. Likewise, the expected frequency of each of the aforementioned archetypal networks will vary between network ensembles, as will the ways in which these subgraphs are embedded into the wider network structure. This makes it a nontrivial question as to how, and indeed whether, the patterns observed for small networks scale up to more realistic network sizes.

**Figure 4:**
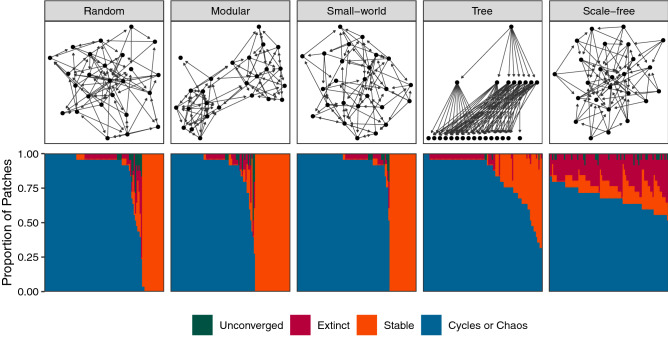
The proportion of patches exhibiting each dynamical regime in each of 100 random networks per network structure ensemble. Each panel shows stacked bar charts, with networks lined up along the horizontal axis, sorted according to the proportion of patches exhibiting oscillatory dynamics. Each bar is colored according to the equilibrium dynamical regime of each of the 25 patches per network. “Extinct” indicates a disease-free equilibrium for that patch, “Stable” indicates a constant prevalence through time, and “Cycles or Chaos” indicates that the prevalence fluctuates through time. “Unconverged” indicates patch dynamics that could not be classified within the timescale of the simulation. For example, looking at the tree networks, every patch in the left-most network exhibited oscillatory dynamics, while the right most network had 18 ($\approx 75\%$) patches exhibiting “Stable” dynamics. Networks were generated according to one of five algorithms (see Methods). Similar results are obtained with alternative parameter values (Supporting Information Fig. S9).

**Figure 5:**
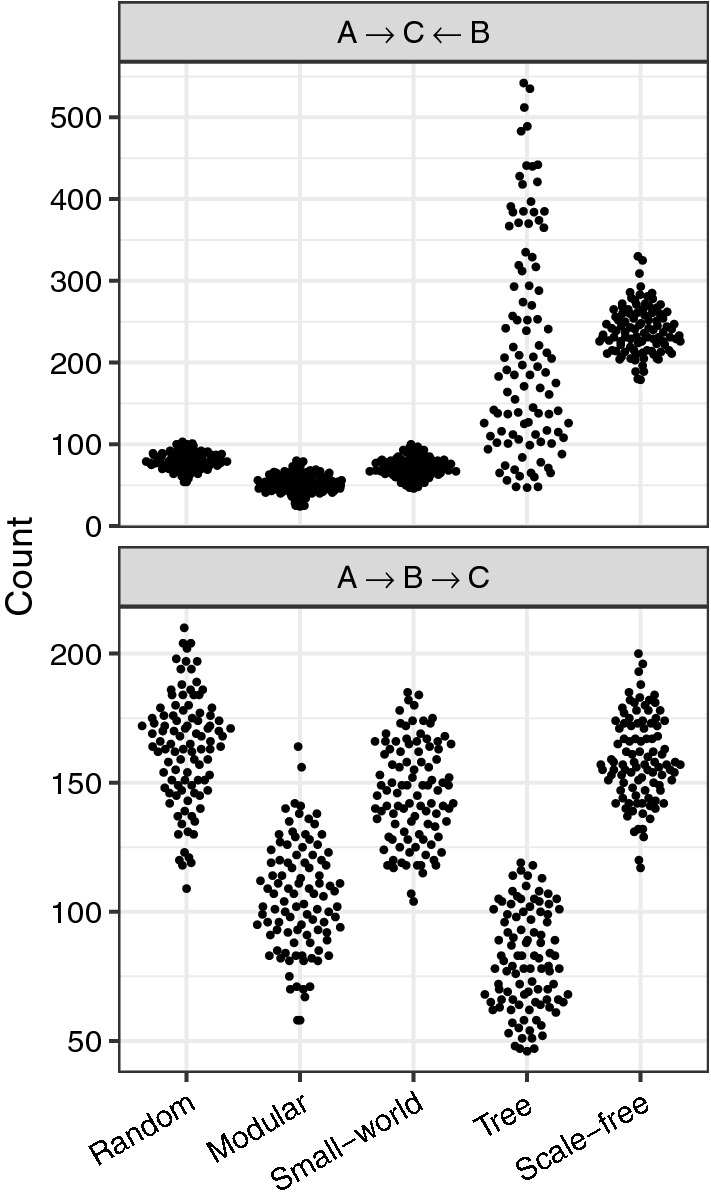
Triad counts for two of the possible configurations of three-node directed subgraphs in each random metapopulation network that correspond to the aforementioned archetypal networks. Points are grouped according to network structure ensemble (see Methods and top row of Fig. 4). As in Fig. 4, we see that for the “in-star” triad ($A \rightarrow C \leftarrow B$), random, modular, and small-world networks tend to have similar values, while tree and scale-free networks differ. In contrast, we see no consistency between differences in the number of chain triads ($A \rightarrow B \rightarrow C$) and the distribution of patch dynamics.

While random, modular, and small-world networks all had similar distributions of dynamics across patches, with most metapopulations consisting of entirely oscillatory or entirely steady-state dynamics, tree and scale-free networks instead tended to show a diversity of dynamics across the metapopulation (Fig. 4). Network structure also varied across network types in terms of the prevalence of three-node subgraphs similar to the archetypal networks considered above. In particular, we note the frequency of in-star triads and three-node chains present in each generated network (Fig. 5). While these metrics consider network structure independent from the dynamics of disease within the composite nodes, such counts can be considered as a proxy for opportunities to observe the patterns noted in Figs. 1 to 3. Tree and scale-free networks tended to have more in-star triads (Fig. 5, again correlating with the differences in disease dynamics observed in Fig. 4 (though variance in indegree (immigration) appears to be an even better predictor of the distribution of dynamics (Supporting Information Fig. S6)).

The findings for these larger networks are in line with our predictions from the archetypal subgraphs considered earlier: as predicted, we see that patches with no immigration tend to have higher pathogen variance than patches that have at least one source of incoming host movement (Mann-Whitney $p < 0.001$), and most metapopulations show a preponderance of oscillatory behavior (Fig. 4). These results also parallel findings of increased epidemic size in scale-free network structures due to the high-degree nodes serving as “super-spreaders” when the overall rate of spread is sufficiently slow^72–75^. Along these lines, there has been some previous research indicating that node degree is directly related to pathogen prevalence in that focal patch^76^, but see^77^. To our knowledge, however, no previous study has considered the distribution of qualitative local dynamical regimes across a metapopulation of more than two patches^48^. A comprehensive investigation of the role of more complex network structures in disease dynamics remains a topic for further investigation.

### Future directions

In probing the relationship between origin and destination dynamics in simple metapopulations, we demonstrated several patterns that expand our understanding of disease dynamics. By directly incorporating a movement network into our model framework, we outline an approach that lends itself to arbitrarily large and complex systems. This is noteworthy, as more and more natural systems are being thought of in terms of networks of interacting components (e.g., separate species in ecological communities^78^ or conspecific host individuals exchanging parasites^79^). By adjusting the scale of our metapopulation, we can ask and answer different questions about the forces influencing disease dynamics. For instance, a metapopulation in which nodes represent countries and edges international travel could shed light on the role of immigration policy on disease dynamics at the national scale^9,17,28,80^. Alternatively, a metapopulation in which the nodes are individuals and edges interpersonal interactions could be used to investigate the interdependence of within-host disease dynamics in relation to sociality^81,82^.

Critically, our results presented here are numerical, rather than analytical. While a full analysis of the mechanisms behind the patterns observed here is still outstanding, a number of mechanisms have been identified for (de)stabilizing population dynamics in prior literature, including spatial and temporal heterogeneity, spatial aggregation, functional response type, etc.^35,36^. Interestingly, in the results presented here, the same superficial change (host movement between patches) can be both stabilizing and destabilizing based solely on the dynamics found in the origin of the movement^48^. Due to the complexity of even moderately sized metapopulations, it is difficult to generalize results from two- or three-patch metapopulations to systems of the size commonly seen in nature. Nevertheless, progress in understanding the precise, mathematical mechanisms behind the changes in dynamics noted in the archetypal cases noted here is essential to make progress on larger, more impactful systems such as the spread of disease across nations, patchy habitats, or livestock production systems.

Finally, while we consider the distribution of dynamical regimes within the network (Fig. 4), we do not explicitly consider the spatial arrangement of these dynamics in relation to one another. Are the oscillatory patches clustered within the network? Do adjacent patches share dynamics more often than would be expected? A full analysis of how dynamical regimes are positioned across network structure, and in relation to the dynamics of nearby patches is a clear next step from these analyses.

### Limitations

Any theoretical study involves simplification, and several of our assumptions can be critiqued as unrealistic. One example is the assumption of continuous movement. While continuous movement might be appropriate for very large patches with frequent, relatively small movements between them, when any of these three components is not present, we would expect deviation from these predictions. Future work could explore the importance of discrete movement regimes on these patterns.

We also use a deterministic model of disease spread. The lack of stochasticity (demographic and environmental) is particularly noteworthy in the context of complex cyclical and chaotic dynamics, where population or pathogen densities occasionally recover from near infinitesimal levels. Such troughs in density are prone to stochastic extinction in real ecosystems^49^. Some previous work on stochastic epidemics on metapopulations has suggested strong correlations in prevalence between connected patches^83^, in line with our findings for connected, identical patches. While the consideration of a stochastic model is beyond the scope of this work, we highlight the need for further exploration of its impact on the patterns described here, and specifically point to the stochastic metapopulation model proposed by^83^ as an avenue for consideration.

Another key assumption is that of unidirectional movement, despite many empirical systems having bidirectional movement (i.e., concurrent movement from $A \rightarrow B$ *and* movement from $B \rightarrow A$). This decision was primarily driven by the underlying theoretical question: how does movement of individuals from one patch to another alter the dynamics in the destination? With bidirectional movement, even identifying which patch is the origin and which is the destination becomes nontrivial. Yet there are also empirical systems in which directional movement is the rule, not the exception, such as in the case of livestock production^84^, riverine metacommunities^85^, or stage-structured populations^35^. Several previous studies have considered bidirectional movement in metapopulation contexts^48,57–60^, finding it to be generally stabilizing when at sufficient levels, a finding we were able to replicate in our system as well (Supporting Information Fig. S2).

Finally, even our “larger” networks are much smaller than the average empirical metapopulation. Further research is needed to explore these patterns in the context of larger and more empirically structured networks.

### Conclusions

We found that the dynamics of pathogen prevalence among patches connected through movement are not independent, and that even very small rates of movement (Supporting Information Fig. S4) can have profound effects on local disease dynamics: from reducing pathogen prevalence to changing the dynamical regime of destination patches entirely. When patches that would exhibit different dynamical regimes are linked, destination patches tend to adopt the dynamics of their origins. Remarkably, given sufficient host movement, this effect is symmetric: oscillatory prevalence can be stabilized by movement from a steady-state patch and the steady-state patch can be driven into cyclical or chaotic behavior if that movement is reversed. Unsurprisingly, the latter is easier to obtain, being both able to persist at lower rates of host movement and dominating over movement from other steady-state populations when there are multiple origin patches at play. Relating the patterns observed in these archetypal patch relationships to differences that arise when considering the structure of larger networks is nontrivial, yet we observed significant differences in the distribution of local pathogen dynamics across network types that correlate with metapopulation structure.

Critically, though we focus only on one underlying disease model^49^ in the main text, our results are fully replicated under a second, independent disease model^50^ in the Supporting Information Section S2. These consistent, replicated findings across parameterizations and even model frameworks suggest these patterns are inherent to pathogen spread on metapopulations, rather than merely an artifact of any particular methodological choice. While the structure of metapopulation networks can be staggeringly complex, the results presented here suggest this complexity may be undergirded with relatively simple patterns of how local disease dynamics become intertwined with one another through host movement.

## Methods

### Model framework

We replicate all simulations using two underlying models of disease spread: a Susceptible-Exposed-Infectious model^49^, and a compartmental model of multi-strain disease^50^. As results are qualitatively similar between model formulations, we present the former here and largely relegate the latter to the Supporting Information (Section S2). Importantly, we view this consistency between models as suggestive of robustness of our results to model formulation. We chose the two models detailed here and in the Supporting Information for their ability to intrinsically exhibit a wide range of dynamical regimes, including pathogen extinction, constant prevalence through time (i.e., a steady-state equilibrium), and fluctuating prevalence through time (i.e., cyclic^49^ or chaotic^50^ attractors).

The compartmental model described by Anderson *et al.*^49^ delineates a population into classes based on their disease status, each class’s dynamics being governed by an ordinary differential equation. Individuals can be either “Susceptible” to infection (*S*), infected but not yet infectious (i.e., “Exposed”; *E*), or “Infectious” (*I*). Infection is assumed to be lifelong and new susceptible individuals are born into the system at a constant per capita rate:

1 $$\begin{aligned} \begin{aligned} \frac{\text {d} S}{\text {d}t}&= r S \left( 1 - \frac{N}{K}\right) - \beta S I\\ \frac{\text {d} E}{\text {d}t}&= \beta S I - (\sigma + \mu + r \frac{N}{K}) E\\ \frac{\text {d} I}{\text {d}t}&= \sigma E - (\nu + \mu + r \frac{N}{K}) I~. \end{aligned} \end{aligned}$$

Note that we have updated the equation lettering to reflect the modern *SIR* framework: replacing the parameter $\gamma$ with the equivalent *r*/*K*, and parameters *b* and $\alpha$ with $\mu$ and $\nu$, respectively. Thus, $N = S + E + I$ is the total host density, *r* is the per capita population growth rate (i.e., the difference between the per capita population birth and death rates), *K* is the host carrying capacity (measured as a density), $\beta$ is the transmission coefficient, $\sigma$ is the inverse of the average latent period, $\mu$ is the per capita death rate, and $\nu$ is the rate of disease-induced mortality. Note that this formulation assumes that only susceptible hosts reproduce.

Following^45^, we modify the homogenous-population disease model using a movement matrix $\varvec{\Delta } = \varvec{X} - \varvec{Y}$, where $\varvec{X}$ a matrix representing immigration, with $X_{ij}$ indicating the rate of movement from patch *i* (row) to patch *j* (column) per unit time and $\varvec{Y}$ is a diagonal matrix representing emigration, where each entry $Y_{ii}=\sum _{j=1}^n X_{ij}$ where *n* is the number of patches. The whole system can thus be depicted by a set of three equations per patch *i*:

2 $$\begin{aligned} \begin{aligned} \frac{\text {d} S_i}{\text {d}t}&= r_i S_i \left( 1 - \frac{N_i}{K_i}\right) - \beta _i S_i I_i + \sum _j \Delta _{ji} S_j\\ \frac{\text {d} E_i}{\text {d}t}&= \beta _i S_i I_i - (\sigma _i + \mu _i + r_i \frac{N_i}{K_i}) E_i + \sum _j \Delta _{ji} E_j\\ \frac{\text {d} I_i}{\text {d}t}&= \sigma _i E_i - (\nu _i + \mu _i + r_i \frac{N_i}{K_i}) I_i + \sum _j \Delta _{ji} I_j \end{aligned} \end{aligned}$$

Each parameter is now indexed according to its patch *i*. In principle, these parameters could vary between patches (e.g., one patch might grow faster than another: $r_i > r_j$), yet, for simplicity of presentation, we keep most parameters constant across the metapopulation, varying only those necessary to alter the dynamical regime between patches. For this, we focus on the patch carrying capacity $K_i$, which is directly associated with the dynamical regime for this system of equations^49^ and is biologically realistic to vary between metapopulation patches. The rate of host movement, i.e., the elements of $\varvec{\Delta }$, might likewise differ for each pair of patches (and indeed for each direction therein) in empirical systems, yet we assume a constant value $\delta$ for each rate of movement, i.e., for each non-zero off-diagonal element of $\varvec{\Delta }$. Sensitivity to this value and the effects of emigration on patch dynamics are explored in the Supporting Information (Figs. S3 to S5). We follow^45^ in assuming there are no births, deaths, or infections during movement between patches.

Finally, the sensitivity of results to our particular choice of parameters was assessed through replication of all results with at least two parameterizations (Supporting Information Figs. S8 and S9 and Section S2).

### Simulation Procedure

As noted above, the dynamical regime exhibited by a pathogen following Eq. (1) is directly related to the host population carrying capacity *K* when all other parameters are held constant^49^, so we restrict parameter differences between patches to differences in carrying capacity and focus on varying the matrix $\varvec{\Delta }$ (i.e., the network of host movement) according to the number and pattern of connections for each patch. Expected dynamics for a homogenous population with no movement are detailed in^49^.

A chain of patches, i.e., $A\rightarrow B\rightarrow C\rightarrow D$, can be depicted with the movement network3We set $\delta = 0.1$ and ask how the dynamics of patches further down the chain (i.e., *B*, *C*, *D*) differ from those of the origin patch (i.e., *A*). Importantly, because we are not using a looping movement chain (in order to maintain an explicit origin and destination for each host movement), there is the possibility of edge effects (i.e., because patch *A* does not have any immigration and patch *D* does not have any emigration). The carrying capacities *K* are set to 15, corresponding to cyclical dynamics in the absence of host movement^49^, while other parameters are set to be approximately equal to the empirical estimates in^49^: $r = 0.5$, $\beta = 80$, $\sigma = 13$, $\mu = 0.5$, and $\nu = 73$, and are the same for all patches.

For patches which differ in their parameters, we consider a system of two patches, identical in all respects other than the their carrying capacity *K*, which is set to either induce a steady-state (i.e., a constant prevalence through time; $K = 5$ in patch *B*) or fluctuating prevalence through time (i.e., cyclical or chaotic dynamics; $K = 15$ in patch *A*; all other parameters as noted above). We then display three potential patterns of connection: no movement, unidirectional movement from *A* to *B* ($A\rightarrow B$), and unidirectional movement from *B* to *A* ($B\rightarrow A$). Specifically, we set the movement networks to be4respectively.

To address the case of multiple origin patches feeding into a single destination patch, we consider a system of three patches: $A\rightarrow C\leftarrow B$, or5where patches *A* and *C* have $K = 5$ (steady-state dynamics), but patch *B* has $K = 15$ (chaotic dynamics); all other parameters as above. In all cases, we assess the dynamics through consideration of the timeseries of disease prevalence, i.e., the proportion of each patch’s population that is currently infected with the pathogen^49^.

#### Larger network structure

Considering larger, more complex metapopulations, we perform 100 simulations for each of five network ensembles. For these simulations, we construct directed networks of an arbitrary size of 25 patches and connectance of approximately 0.15, but with varying network structure, according to five random-network ensembles: Erdős-Rényi-Gilbert (links randomly assigned between patches; “random”), stochastic block (two, densely connected “modules” of patches, with few inter-group connections; “modular”), Watts-Strogatz (small-world network structure produced by partially re-wiring a spatial grid of patches; “small-world”), tree-like (many chains of patches and no potential for loops, “tree”), and Barabási-Albert (scale-free degree distribution where few patches have very many links, and many patches have few links; “scale-free”). Note that we use terms like “scale-free” and “small-world” here as short-hand, bearing in mind that such structural generalizations are typically only defined in the limit of much larger network size. Network-generating algorithms from the tidygraph R package^86^ were used, except for tree and Watts-Strogatz configurations which required custom algorithms. Each movement rate was set to $\delta = 0.01$, and each patch was assigned the same disease parameters as above except for carrying capacity and initial densities of susceptible, exposed, and infectious individuals, which are randomized for each patch: $K_i = [5, 20]$ and $X_i(0) = [0, 1]$, where $X \in \{S, E, I\}$ and [*a*, *b*] indicates a uniformly sampled random value between *a* and *b*, inclusive.

For each network, we sought to relate properties of the network structure to the outcomes of simulated disease spread across the metapopulation. For the former, we quantified both properties of each network’s degree distribution (Supporting Information Fig. S6) and the frequency of three-node subgraphs found in each network that form short chains ($A \rightarrow B \rightarrow C$; similar to the network in Eq. (3)) or in-stars ($A \rightarrow C \leftarrow B$; as used in Eq. (5)). To quantify disease outcomes, we simulated 10,000 time-steps of disease spread on each network and A) summarized pathogen prevalence over time (Supporting Information Figs. S7 and S8), and B) categorized the dynamical regime of each patch over the final 1000 time-steps as disease-free (“Extinct”), stable pathogen prevalence (“Stable”), or fluctuating pathogen prevalence through time (“Cycles or Chaos”). Patches which could not be classified into one of these three categories within the timescale of the simulation were labeled “Unconverged” (Fig. 4, Supporting Information Fig. S9).

All numerical integrations were carried out using the DifferentialEquations package^87,88^ in Julia version 1.7.0^89^, with graphics produced using the ggplot2 package^90^ in R version 4.0.3^91^. Code can be found on GitHub: https://github.com/mjsmith037/metapop_local_dynamics.

## Supplementary information

Supplementary Information.
