# Supplementary material for: Asymmetric host movement reshapes local disease dynamics in metapopulations
Source: Sci Rep. 2022 Jun 7;12:9365. doi: 10.1038/s41598-022-12774-5 (PMC9171740; doi:10.1038/s41598-022-12774-5)
Supplement: Supplementary file 1 — Supplementary Information. [file 41598_2022_12774_MOESM1_ESM.pdf]

# Supporting Information to Asymmetric host movement reshapes local disease dynamics in metapopulations

**Matthew Michalska-Smith[1,2,*], Kimberly VanderWaal[1], and Meggan E Craft[1,3]**

[1]Department of Veterinary Population Medicine, University of Minnesota, St. Paul, MN USA
[2]Department of Plant Pathology, University of Minnesota, St. Paul MN, USA
[3]Department of Ecology, Evolution, and Behavior, University of Minnesota, St. Paul MN, USA
[*]Michalska-Smith@pm.me

s

## 1 Additional figures referenced in the main text

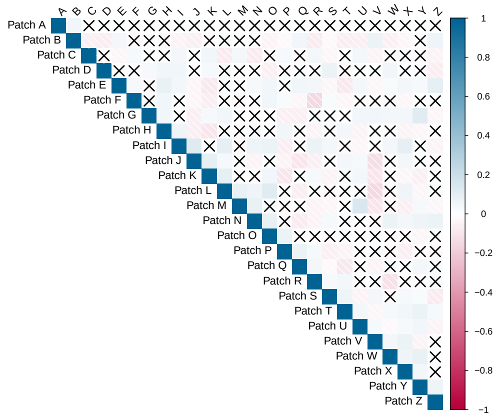

**(a)** Movement rate $\delta = 0.01$

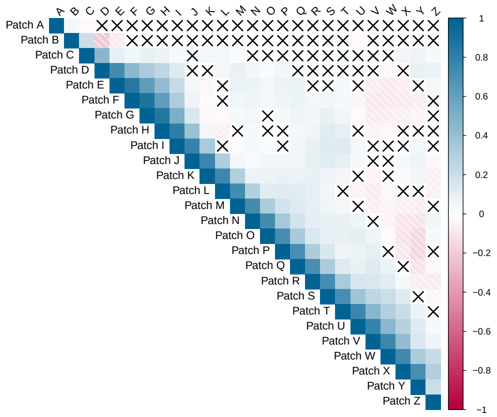

**(b)** Movement rate $\delta = 0.05$

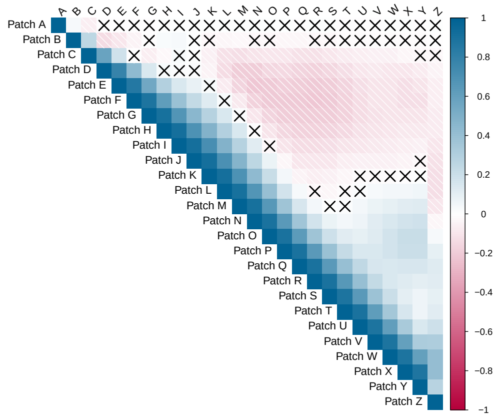

**(c)** Movement rate $\delta = 0.1$

**Figure 1.** Correlograms of each patch's pathogen prevalence through time. Using a metapopulation as in Figure 1, but with a much longer chain of patches, here we plot the correlation between disease prevalence between each pair of patches for the final 10,000 time steps of a 15,000 time step simulation. Reading across the rows shows each patch's correlation with those patches further down the chain. Significant correlations are colored according to their direction (red for negative, blue for positive) and strength, with non-significant correlations indicted by a black X. As expected, correlation with patch $A$ is limited, due to the $A$ being the only patch without any immigration. The extent of correlation between subsequent patches depends on the movement rate.

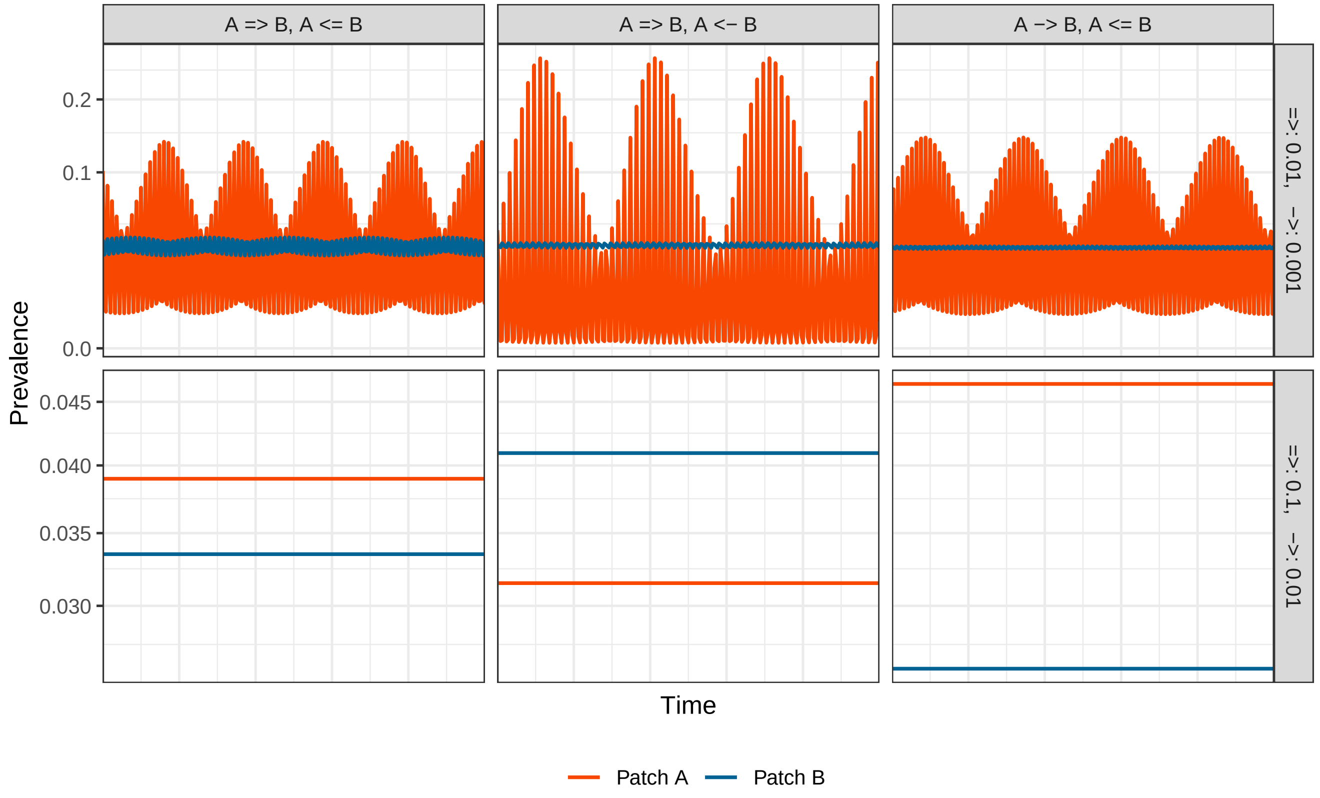


**Figure 2.** Prevalence through time in each patch of a two-patch metapopulation, connected by bidirectional movement, i.e., $A \rightarrow B$ and $B \rightarrow A$. In the left column, the rates of host movement are symmetric, in the middle, the rate from $B \rightarrow A$ is lessened by a factor of 1/10, and on the right, the rate from $A \rightarrow B$ is lessened by a factor of 1/10. Note that the effects of bidirectional movement are less consistent than the case of unidirectional movement shown in Figure 2. Patches are parameterized as elsewhere: $r = 0.5$, $\beta = 80$, $\sigma = 10$, $\mu = 0.5$, and $\nu = 73$.

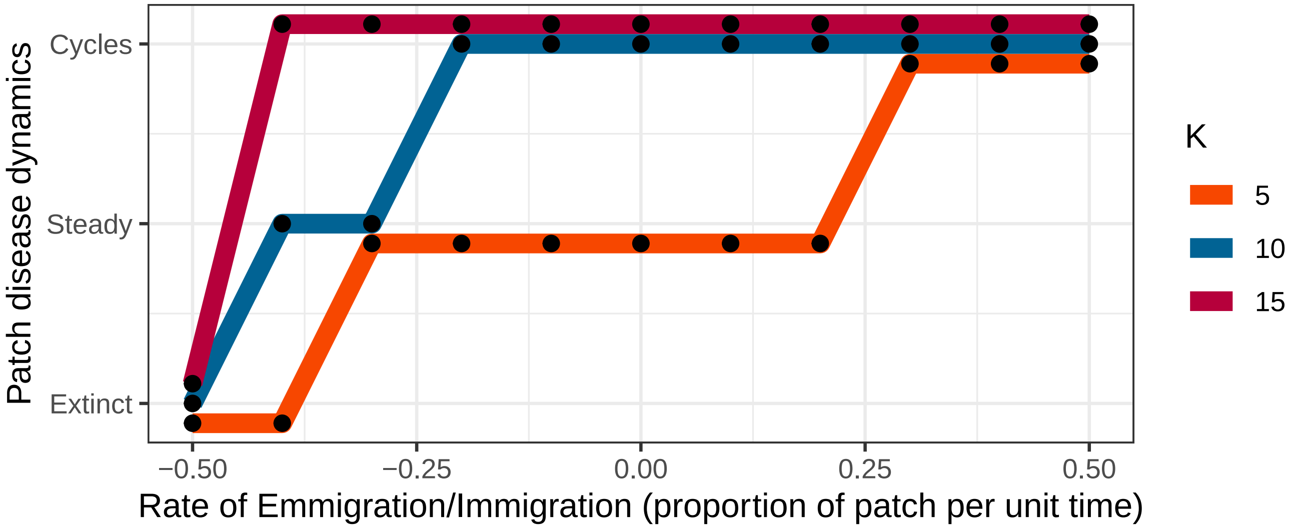


**Figure 3.** Subway diagram showing the effect of various rates of (self) immigration and emigration on local patch dynamics. For these simulations we add a constant $\delta$ value in a single-patch metapopulation. This value can be thought of as either emigration (a negative value for $\delta$) or immigration (a positive $\delta$), but is more precisely an increased death or birth rate, respectively. A value of 0 (center of the plot) corresponds to the single-patch dynamics (i.e., those described by Equation 1). The vertical axis indicates what dynamical regime is being exhibited by the infectious subset of the population: cyclical or chaotic dynamics (hereafter referred to just as "cycles" for simplicity), "steady"-state dynamics, or a disease-free equilibrium "extinct"). As the movement rate ($\delta$; horizontal axis) becomes more negative, dynamics are stabilized (cycles to steady-states) before being driven extinct. As $\delta$ becomes more positive, dynamics are destabilized (steady-states to cycles). Importantly, both of these transitions occur with much larger absolute rates of movement than the transitions described in the main text by connecting patches of different dynamical regimes together (see also Fig. 4). Three values of the carrying capacity ($K$) are recorded here (5, 10, and 15), with additional parameters: $r = 0.5$, $\beta = 80$, $\sigma = 10$, $\mu = 0.5$, and $\nu = 73$.

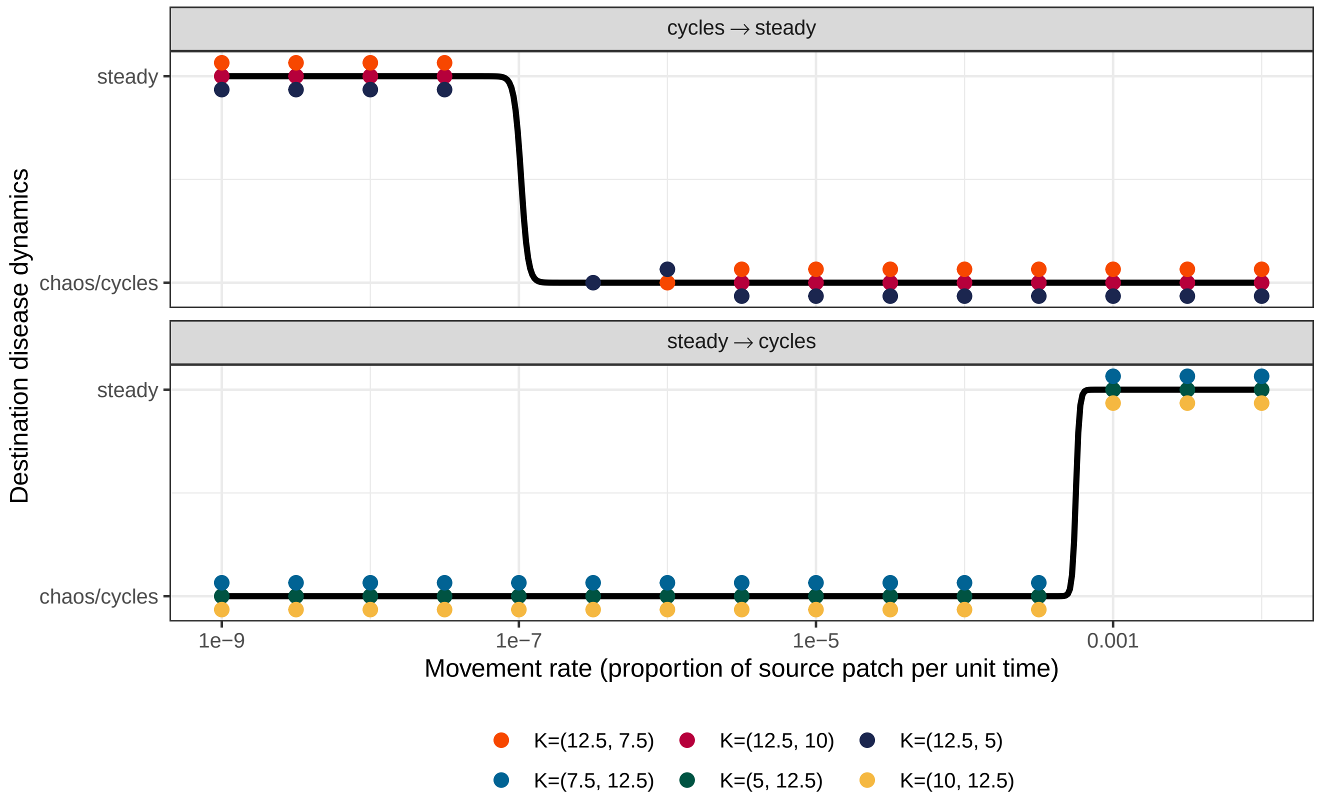


**Figure 4.** The effect of various movement rates on transference of dynamical regime from origin to destination in a simple metapopulation of two patches linked by unidirectional movement. In the top panel are cases where the origin patch has cyclical or chaotic dynamics (hereafter referred to just as "cycles" for simplicity) and the destination has steady-state dynamics when in isolation, while in the bottom panel the opposite is true. As the movement rate ($\delta$; horizontal axis) increases, there is a phase-transition at which point the destination patch's dynamics (indicated by the vertical axis) switch to match those of the origin. We fit a binomial spline to highlight this transition point. We see that even with very small rates of movement, a steady-state patch can be converted to cycles (top panel). Yet, it is more difficult to convert cycles to steady-state dynamics (bottom panel). Three parameterizations are recorded here (each with movement between the patches in two directions), with additional parameters: $r = 0.5$, $\beta = 80$, $\sigma = 10$, $\mu = 0.5$, and $\nu = 73$. Finally, note that the two values of $K$ listed in the legend correspond to the origin and destination patches, respectively.

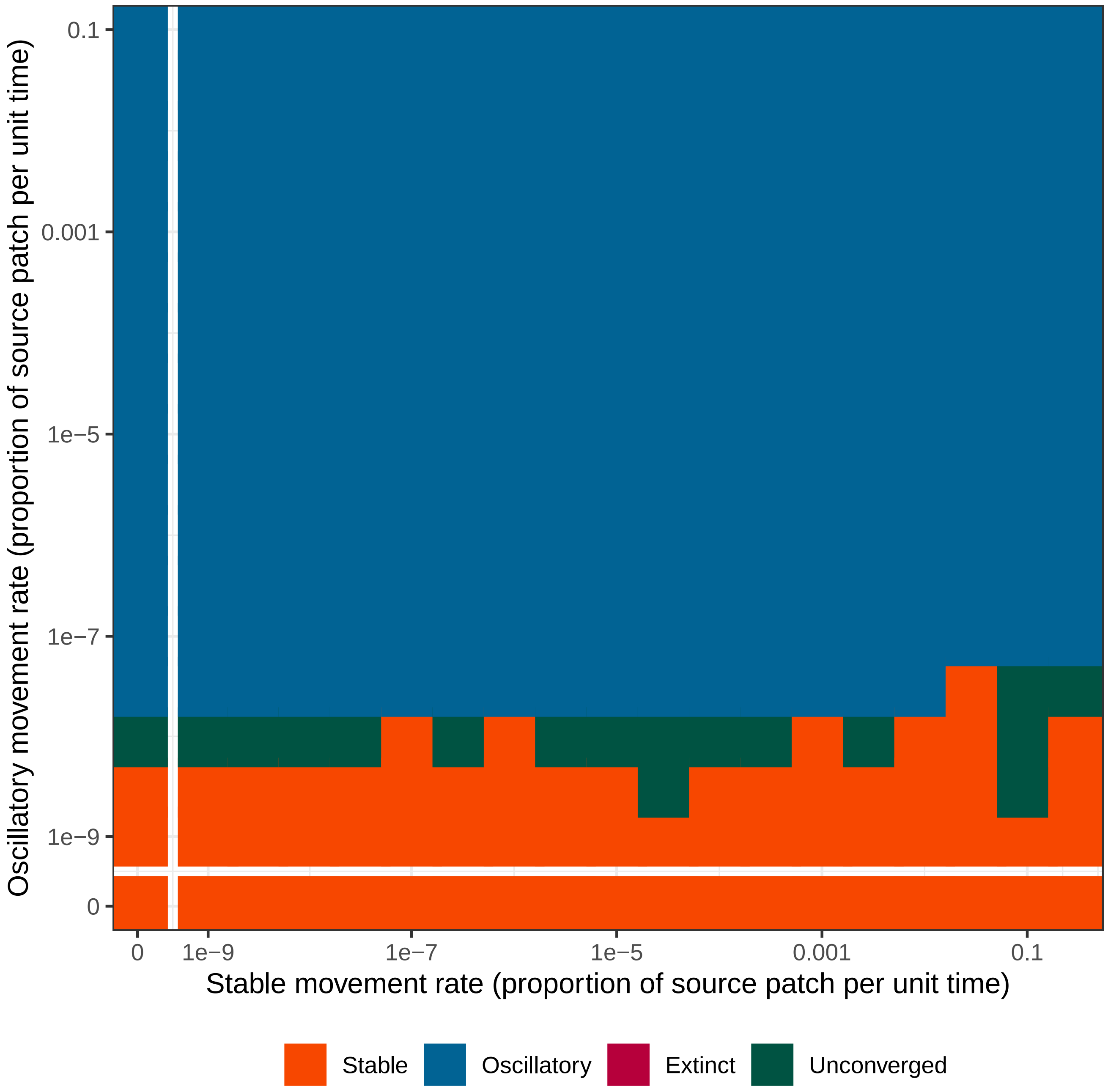


**Figure 5.** The effect of various movement rates on the hierarchical transference of dynamical regime from origin to destination in a simple metapopulation of three patches linked by unidirectional movement. The horizontal axis denotes the movement rate (in proportion of origin patch per unit time) from a patch exhibiting steady-state dynamics, while the vertical axis denotes the movement rate from a patch exhibiting oscillatory dynamics. The destination patch is parameterized to exhibit steady-state dynamics in the absence of host movement. Other than movement rate, all patches are parameterized identically: $r = 0.5$, $\beta = 80$, $\sigma = 10$, $\mu = 0.5$, and $\nu = 73$. Note that, largely independent of the degree of movement from the stable patch, the destination will exhibit oscillatory dynamics in the presence of sufficient movement from the oscillatory patch.

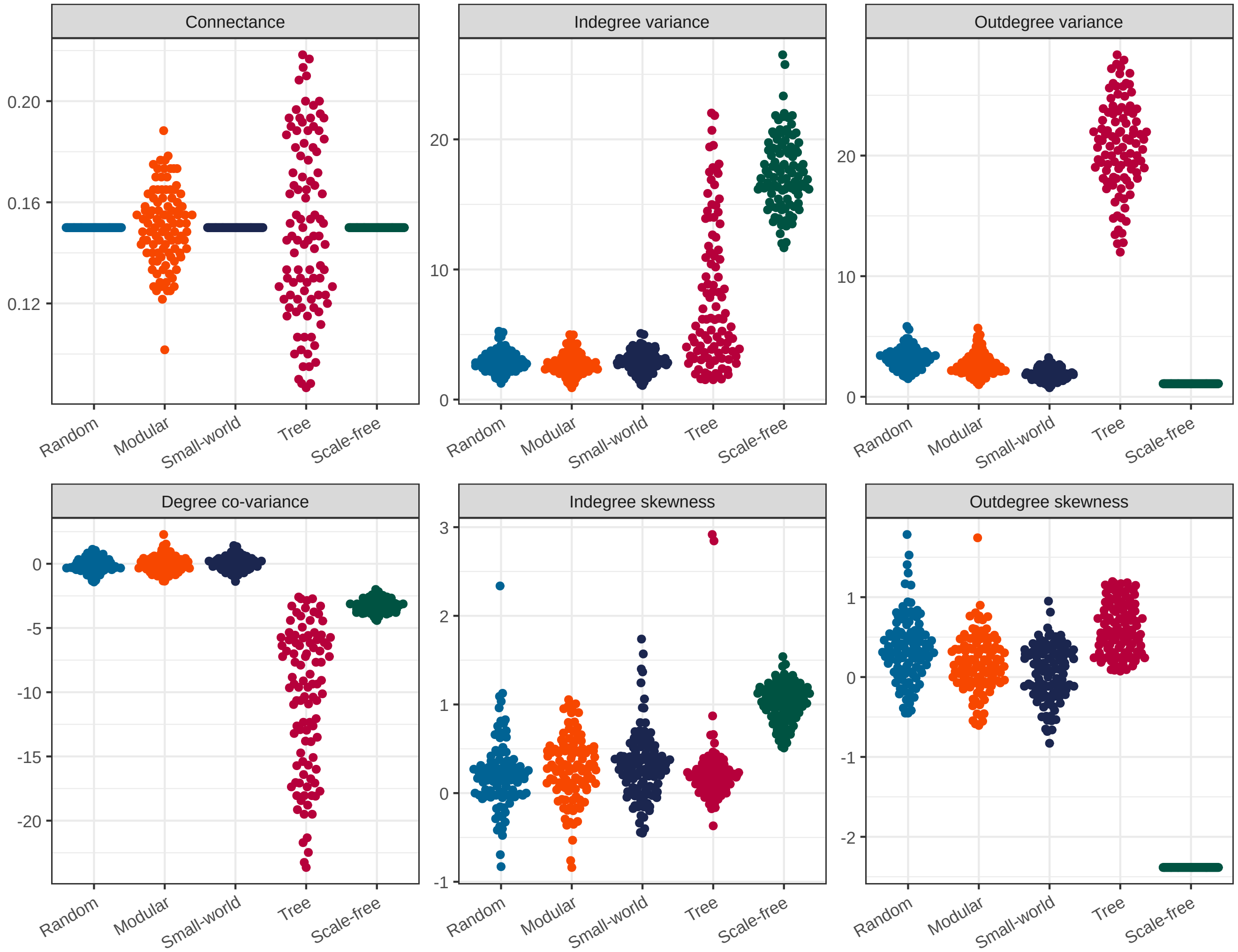


**Figure 6.** Summary statistics for the degree distributions of each randomized network used for Figure 4 in the main text. Networks were constructed to have the same size and approximate connectance, but with the network structure (which patches are connected to which other patches) otherwise generated according to one of five algorithms: Erds-Rényi-Gilbert (“random”), stochastic block (“modular”), Watts-Strogatz (“small-world”), tree, and Barabási-Albert (“scale-free”) (see Methods in the main text). Some algorithms allowed perfect matching of connectance (random, small-world, and scale-free), while others necessitated some variation (modular and tree).

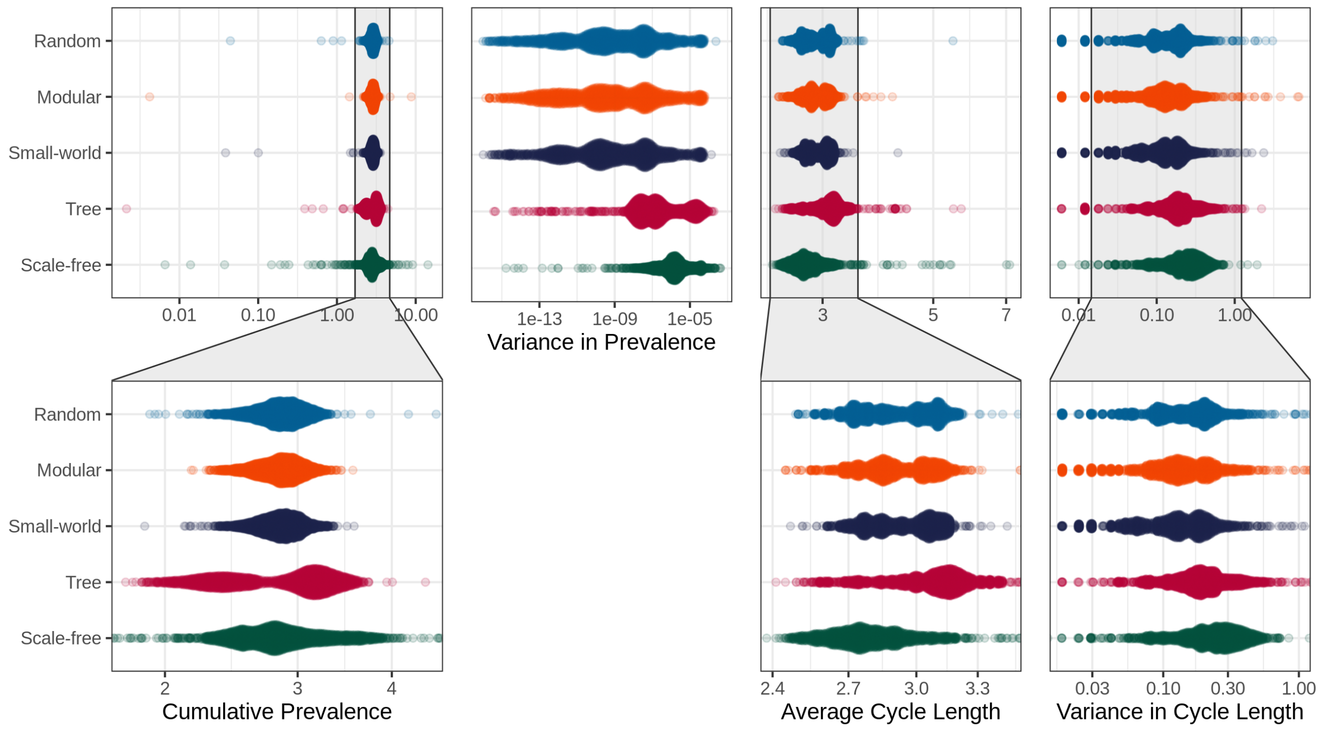


**Figure 7.** The effect of network structure on pathogen prevalence through time. In the top row, we show the distributions of each of four response variables: the total density of infected individuals by the end of the simulation (“Cumulative Prevalence”), the variance in the density of infected individuals through time (“Variance in Prevalence”), the average time between peaks in prevalence (“Average Cycle Length”), and the variance in the times between prevalence peaks (“Variance in Cycle Length;” indicated on horizontal axis labels). The latter two metrics were only measured on patches exhibiting cyclical or chaotic dynamics. The bottom row shows restricted axis limits for a subset of the response variables in order to highlight differences between network distributions. Each distribution is made up of (at most) one point for each of the 25 patches in each of 100 random networks (maximum of 2500 points per distribution). These results are from the same simulations noted in Figure 4. Similar results are obtained with alternative parameter values (Fig. 8).

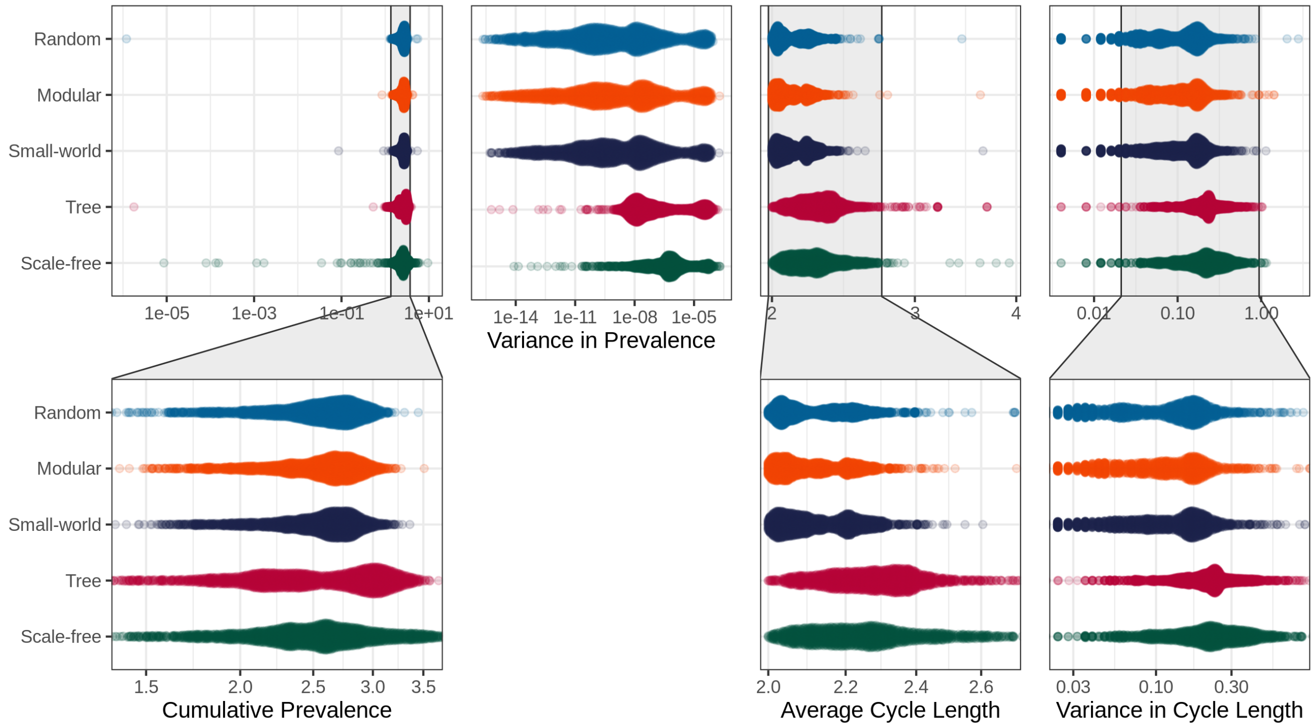


**Figure 8.** As Fig. 7, but with $\sigma = 40$ and $K_i$ sampled from $[2, 10]$. All other parameters are equal to or set randomly as in Fig. 7.

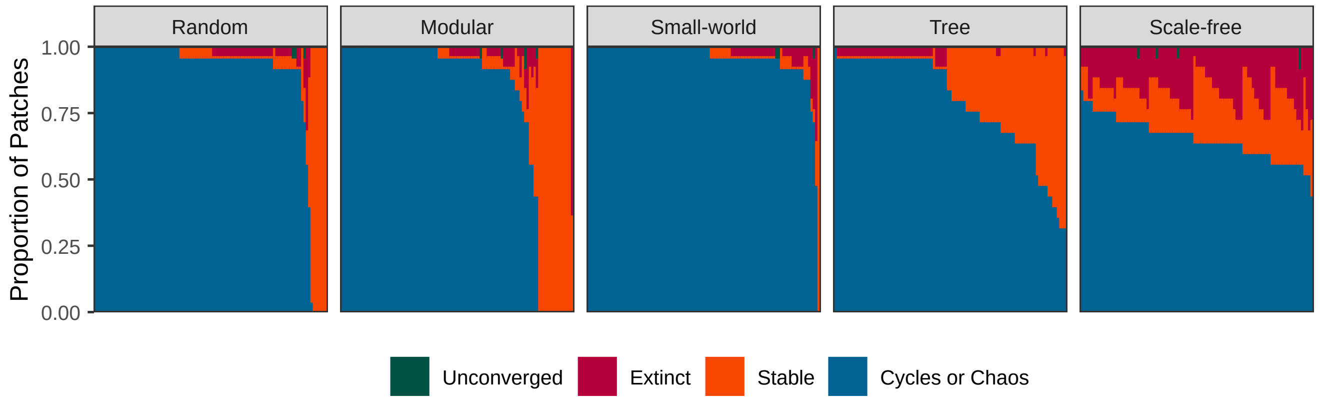


**Figure 9.** As Figure 4, but with $\sigma = 40$ and $K_i$ sampled from $[2, 10]$. All other parameters are equal to or set randomly as in Figure 4.

## 2 Alternative Disease Model

In this section, we replicate the results from above using a multi-strain disease framework developed by[1] and[2].

### 2.1 Model details

This multi-strain model is based on a finite antigenic strain space in which each strain is defined by its combination of “alleles” at a number of “loci.” More concretely, a strain space might be defined by two loci, the first of which possessing three possible alleles (e.g., $A$, $B$, $C$) and the second only two (e.g., $a$, $b$). We can represent this strain space as $\{3,2\}$, where the length of the vector is the number of loci, and the elements indicate the number of potential alleles at each loci. Any particular strain can be represented by a vector of the same length, with values signifying which allele is present at each locus. Thus, an individual can contract any of six possible strains: $\{A,a\}$, $\{A,b\}$, $\{B,a\}$, $\{B,b\}$, $\{C,a\}$, and $\{C,b\}$.

We assume the potential for (partial) cross-reactive immunity when an individual is exposed to a strain that shares at least one allele with strains that that individual has seen previously. For example, if an individual was previously exposed to strain $\{A,b\}$, they can have at least partial immunity to future exposure to any strain containing alleles $A$ or $b$ (i.e., $\{A,a\}$, $\{A,b\}$, $\{B,b\}$, and $\{C,b\}$). In general, we assume complete immunity to future exposure to the exact strains that have been seen before (i.e., $\{A,b\}$; see below).

With that foundation, the model framework is composed of three equations for each strain $i$,

$$\begin{aligned}
\frac{\mathrm{d}y_i}{\mathrm{d}t} &= \beta_i\left((1-w_i)+(1-\gamma)(w_i-z_i)\right)y_i - \sigma_i y_i - \mu_i y_i \\
\frac{\mathrm{d}z_i}{\mathrm{d}t} &= \beta_i(1-z_i)y_i - \mu_i z_i \\
\frac{\mathrm{d}w_i}{\mathrm{d}t} &= \beta_i(1-w_i)\sum_{j\ni(j\cap i\neq\emptyset)} y_j - \mu_i w_i
\end{aligned} \quad . \qquad (1)$$

Note that we index each demographic parameter according to its associated strain. While, in principle, each strain could have a different infection, recovery, or death rate, in the following figures we assume these values are consistent across strains. Put another way, we assume that all strains are functionally equivalent.

Each equation records the proportion of a patch that falls within one of three states with regard to the focal strain $i$: $y$ indicates the proportion of the patch currently infectious with strain $i$, $z$ indicates the proportion of the patch that is immune to further infection with strain $i$, and $w$ indicates the proportion of the patch that has at least partial cross-protective immunity to strain $i$. Note that these equations are nested: individuals that have been exposed to strain $i$ thus have not only partial immunity (i.e., are counted in $w$), but indeed have complete immunity to repeated infection with strain $i$ (i.e., are also counted in $z$). This nesting implies $y_i \leq z_i \leq w_i \;\; \forall\, i$.

Dissecting the $y$ equation, we see that there are standard recovery (rate determined by $\sigma_i$) and mortality (rate determined by $\mu_i$) terms. The infection term, however, warrants further explanation. Consider that the proportion of the patch that is susceptible to infection with strain $i$ includes all individuals who do not already have complete immunity to it, i.e., $1-z_i$. This proportion can be divided into those who also lack partial immunity $1-w_i$ and those that have partial, but not complete immunity $w_i - z_i$. While the former lacks any immunity and is thus infected simply at a rate $\beta_i$, the latter has its infection rate attenuated by a proportion $0 \leq \gamma \leq 1$. Put another way, individuals who had previously been exposed to related strain $j$ have a $\beta(1-\gamma)$ rate of infection with strain $i$, rather than the straight $\beta$ rate experienced by individuals who had not seen $j$ (or another related strain) previously.

The $z$ equation contains only two parts: mortality (governed by the parameter $\mu_i$, as above), and exposure. Note that we do not say “infection” here. While one might expect a division of susceptible individuals into those that are completely naive and those that have partial immunity as was seen in the $y$ equations, instead we just use $1-z_i$ in the $z$ equations. This is because an individual gains specific (i.e., complete) immunity to future infection *both* in the case of infection from a completely naive state $\beta_i(1-w_i)y_i$ and in the case of exposure to strain $i$ when partial (but not complete) immunity is already present $\beta_i(w_i-z_i)y_i$. When added together, these can be represented more simply as just $\beta_i(1-z_i)y_i$. Put another way, individuals that are exposed to similar strain $j$ (following infection with strain $i$) will enter the $z_j$ class *whether or not* they enter the $y_j$ class. Importantly, this means that the cross-protective immunity $\gamma$ reduces the likelihood of individuals becoming infectious, but it does not reduce their rate of obtaining immunity to additional, related strains.

Finally, the $w$ equation also contains mortality ($\mu_i$, as above) and exposure. As with the specific immunity gained in the $z$ equation, the gaining of partial immunity requires only that an individual without partial immunity be exposed to an individual infectious with a strain $j$ that contains at least one allele in common with the focal strain $i$. The exposures across strains are summed to get the total increase in partial immunity over time. Note that this is the only part of the equations that integrates

information across strains and thus serves as the foundation for the complex strain-competition dynamics that have been noted previously[1–3] and in this work.

Analogously to the modifications of the[4] model in the main text, to incorporate a metapopulation structure, we add a term to each of the equations above corresponding to host movement:

$$\begin{aligned}\frac{\mathrm{d}y_i}{\mathrm{d}t} &= \beta_i\left((1-w_i)+(1-\gamma)(w_i-z_i)\right)y_i - \sigma_i y_i - \mu_i y_i + \sum_j \Delta_{ji} y_j \\ \frac{\mathrm{d}z_i}{\mathrm{d}t} &= \beta_i(1-z_i)y_i - \mu_i z_i + \sum_j \Delta_{ji} z_j \\ \frac{\mathrm{d}w_i}{\mathrm{d}t} &= \beta_i(1-w_i)\sum_{j\ni(j\cap i\neq\emptyset)} y_j - \mu_i w_i + \sum_j \Delta_{ji} w_j\end{aligned} \quad . \tag{2}$$

Where $\mathbf{\Delta}$ is defined as in the main text, with each off-diagonal element $\Delta_{ij} = \delta$ set to a small constant indicating the proportional rate at which a given class in patch $i$ that moves to patch $j$.

Note that, in contrast to[1], we use $\beta$ to regulate the dynamical regime in each patch, rather than $\gamma$. We found this to be a more biologically realistic parameter to differ between patches, e.g., due to nonlinear effects of population density.

## 2.2 Connecting patches with the same dynamics

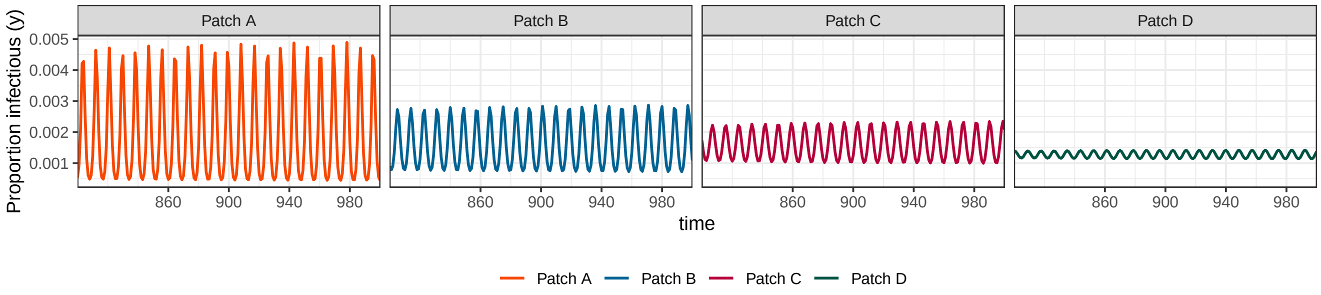


**Figure 10.** Connecting multiple patches with the same parameters results in reduced pathogen prevalence and dampened oscillations in patches further down the chain. Here, patches are connected such that $A \to B \to C \to D$. As in Figure 1 in the main text, each panel indicates a patch, and the vertical axis shows the proportion of each patch that is currently infectious (equation $y$ from above). All patches have parameters $\sigma = 8$, $\mu = 0.1$, $\delta = 0.052$, $\gamma = 0.66$ and a $\beta = 16$ for all patches. We use a three-loci, two-allele strain structure, but show only one strain for clarity (but see Fig. 15). Likewise, transient dynamics are omitted from the time series for clarity.

## 2.3 Connecting patches with differing dynamics

### *2.3.1 Dynamics Propagation*

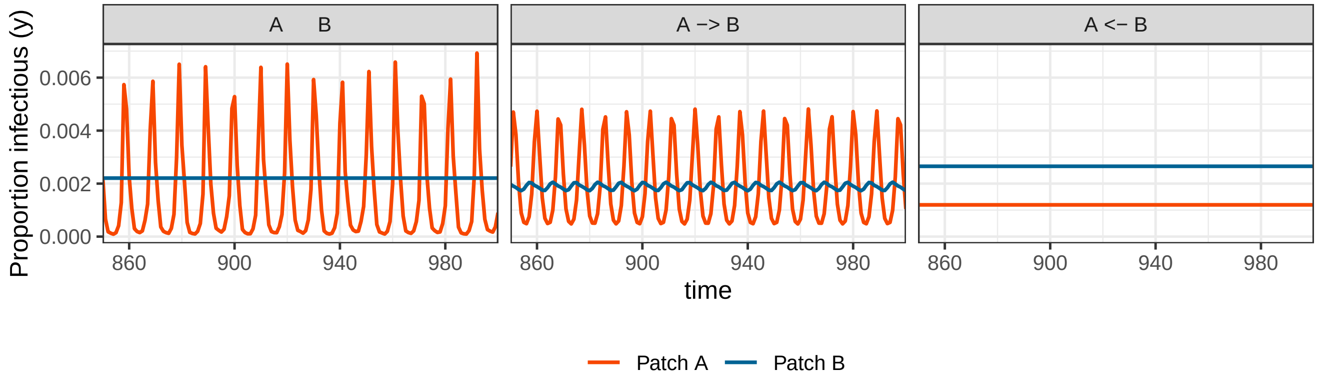


**Figure 11.** Destination patches tend to inherit origin patch dynamics when linking patches with different model parameterizations. Axes are as in Fig. 10, but here panels indicate network structure. While in isolation (left panel), patch $A$ has cyclical dynamics and patch $B$ has steady-state dynamics, when the two patches are linked by movement, the destination patch inherits the dynamics of the origin patch (center and right panels). This is true regardless of the direction of the movement (depending on the rate of movement; Fig. 16). Patches $A$ and $B$ have parameters $\sigma = 8$, $\mu = 0.1$, $\delta = 0.05$, and $\gamma = 0.66$ in common and $\beta = \{16, 40\}$, respectively. We use a three-loci, two-allele strain structure, but show only one strain for clarity. Likewise, transient dynamics are omitted from the time series for clarity.

### *2.3.2 Dynamics Hierarchy*

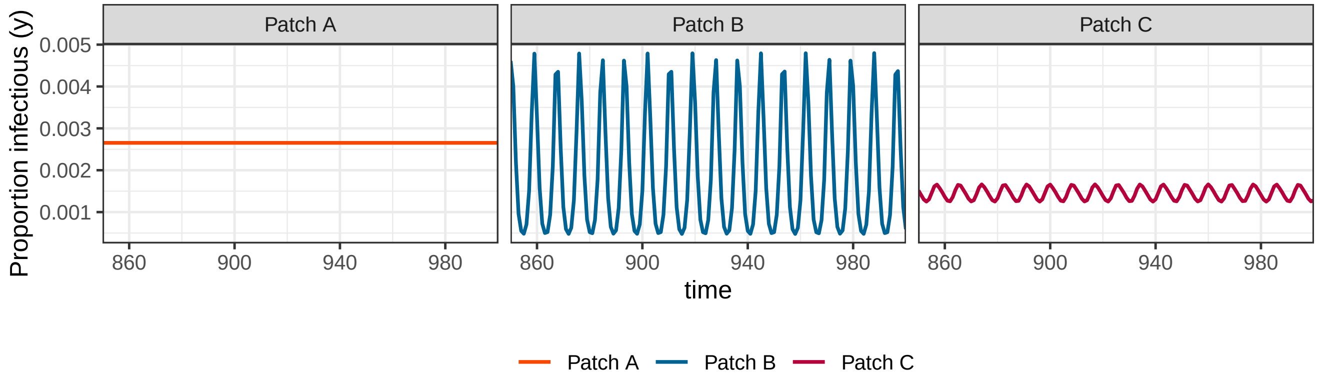


**Figure 12.** When multiple origin patches differ in their dynamics, the destination patch inherits oscillations over steady-states. Axes and panels are as in Fig. 10. Here, we have patches $A$ and $B$ feeding into patch $C$ at the same rate of $\delta = 0.05$. Patches $A$ and $C$ are parametrized to produce steady-state dynamics in the absence of movement, with $\sigma = 8$, $\mu = 0.1$, $\gamma = 0.66$, and $\beta = 16$. Patch $B$ shows cyclical dynamics with $\beta = 40$ and all other parameters the same. Note that, even though the parameters of patch $C$ would lead to a steady-state in the absence of movement, we see cyclical dynamics being inherited from patch $B$. We use a three-loci, two-allele strain structure, but show only one strain for clarity. Likewise, transient dynamics are omitted from the time series for clarity.

## 2.4 Generalizing to Larger Networks

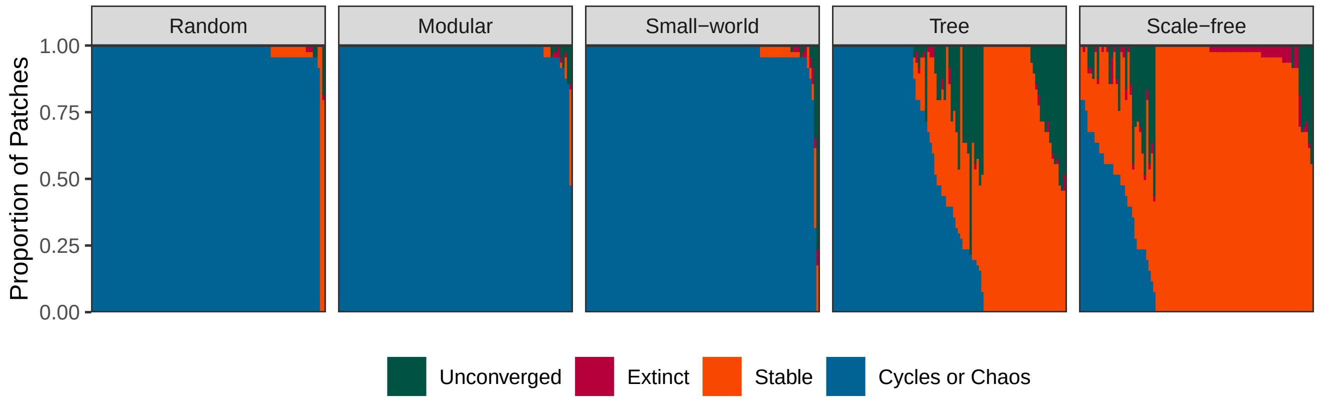


**Figure 13.** The proportion of patches exhibiting each dynamical regime in each of 100 random networks per network structure ensemble. Each panel shows stacked bar charts, with networks lined up along the horizontal axis, sorted according to the proportion of patches exhibiting cyclical dynamics. Each bar is colored according to the equilibrium dynamical regime of each of the 25 patches per network. “Extinct” indicates a disease-free equilibrium for that patch, “Stable” indicates a constant prevalence through time, and “Cycles or Chaos” indicates that the prevalence fluctuates through time. “Unconverged” indicates patch dynamics that could not be classified within the timescale of the simulation. Model parameters were either the same for all patches and across simulations ($\sigma = 8$, $\mu = 0.05$, $\delta = 0.01$, and $\gamma = 0.66$) or randomized for each patch in each simulation (initial densities of infectious and immune individuals $[0,1]$ and $\beta = [16,48]$ for each patch). Similar results are obtained with alternative parameter values (Fig. 17).

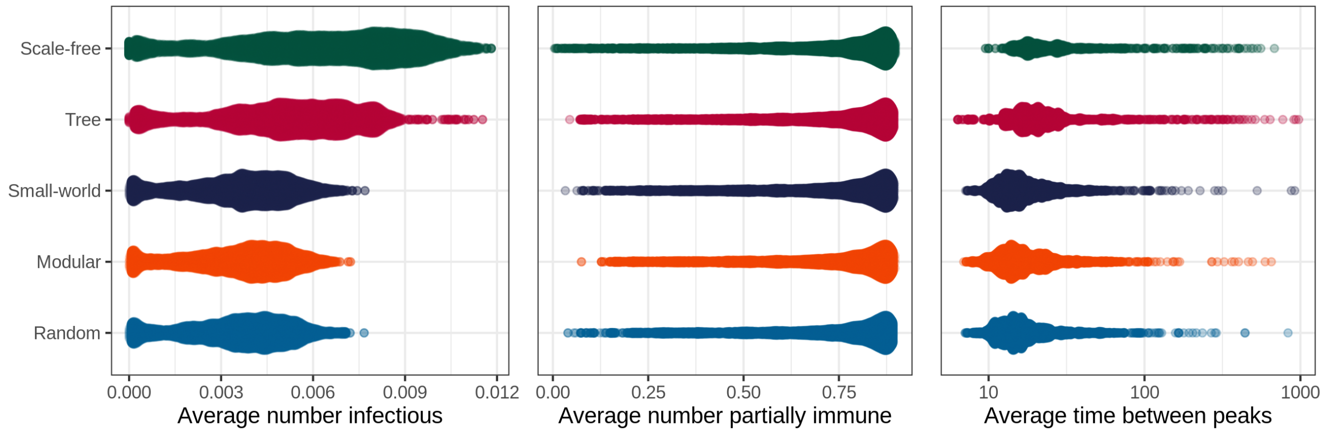


**Figure 14.** The effect of network structure on pathogen prevalence and levels of immunity through time. Each panel shows the distributions of one response variable for prevalence of the pathogen and specific immunity over the course of the simulation. We depict one point for each randomized network structure and box-plots indicating the median and inter-quartile range of each network-type’s distribution. Network generating algorithms were tuned to produce networks of the same size and approximate connectance and model parameters were either the same for all patches and across simulations ($\sigma = 8$, $\mu = 0.05$, $\delta = 0.01$, and $\gamma = 0.66$) or randomized for each patch in each simulation (initial densities of infectious and immune individuals $[0,1]$ and $\beta = [16,48]$. Similar results are obtained with alternative parameter values (Fig. 18).

## 2.5 Additional figures

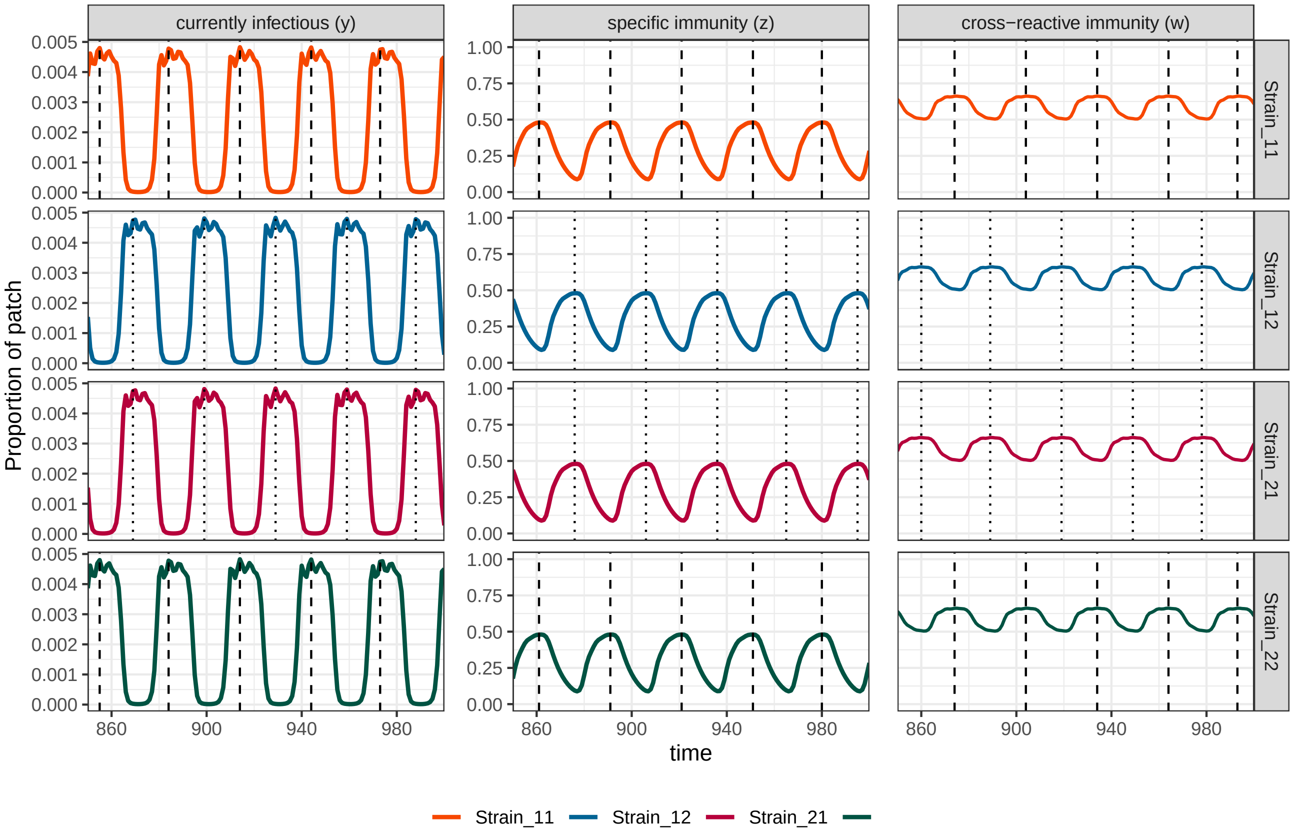


**Figure 15.** The relationship between strains in the multi-strain framework of[1]. Note that lines are colored according to strain rather than patch. In a two-loci, two-allele strain structure, strains can be divided into two discordant sets of non-overlapping alleles: {1, 1} and {2, 2}, and {1, 2} and {2, 1}. Each strain of a discordant set behaves identically due to identical parameterization and no interaction between strains that do not share at least one allele, but discordant sets interact with one another due to partial cross-reactive immunity. Thus, when one set is abundant, the other is rare and *vice versa*. We highlight the maximum value of each discordant set's cycle with a vertical line in order to facilitate comparisons between strains and sets. Transient dynamics are omitted from the time series for clarity.

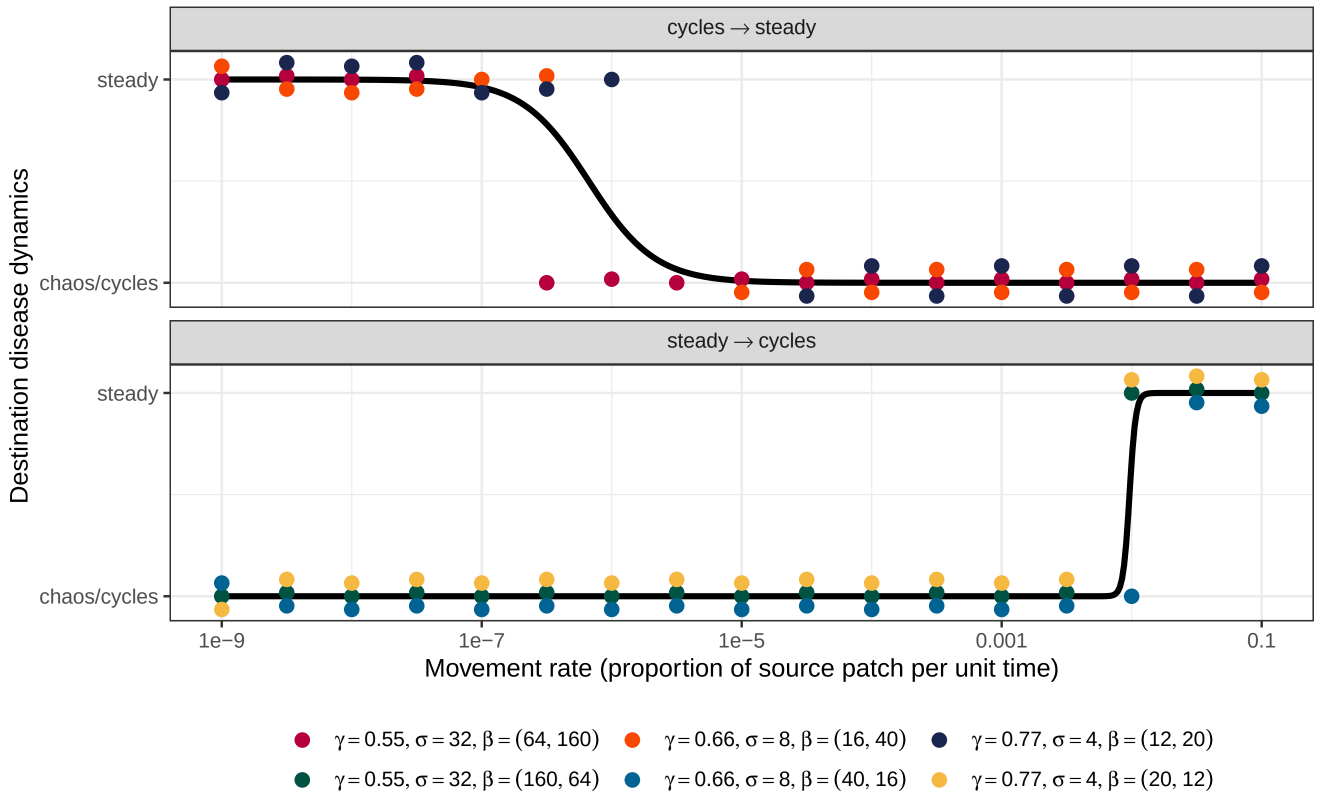


**Figure 16.** The effect of variable movement rate on transference of dynamical regime from origin to destination in a simple metapopulation of two patches linked by unidirectional movement. In the top panel are cases where the origin patch has cyclical dynamics and the destination has steady-state dynamics when in isolation, while in the bottom panel the opposite is true. As the movement rate ($\delta$; horizontal axis) increases, there is a phase-transition at which point the destination patch's dynamics (indicated by the vertical axis) switch to match those of the origin. We fit a binomial spline to highlight this transition point. We see that even with very small rates of movement, a steady-state patch can be converted to a cyclical one (top panel). Yet, it is more difficult to convert a cyclical patch to one with steady-state dynamics (bottom panel). Three parameterizations are recorded here (each with movement between the patches in two directions), with additional parameter $\mu = 0.15$ being the same for both patches. Finally, note that the two values of $\beta$ listed in the legend correspond to the two patches, with the larger value corresponding to the steady-state patch and the smaller value the cyclical patch (when in isolation).

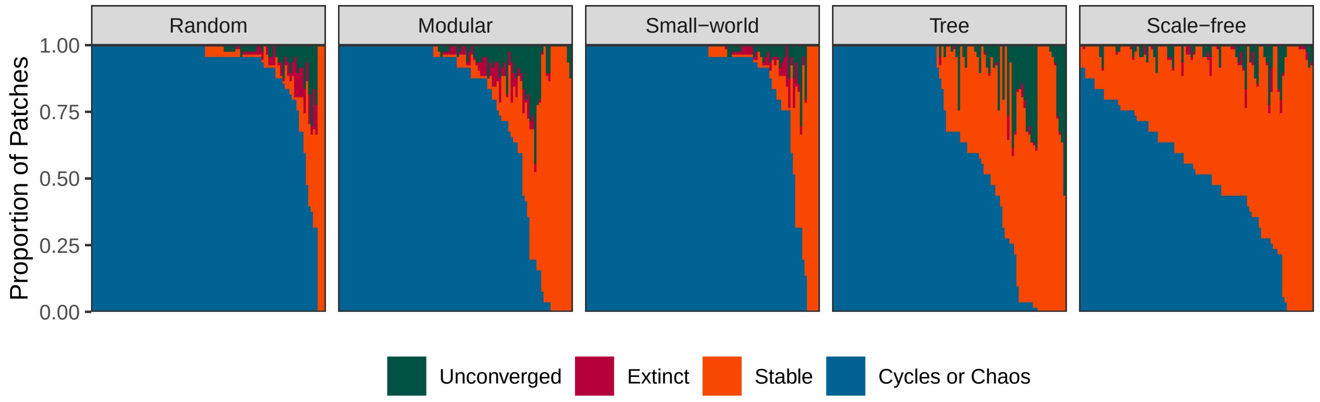


**Figure 17.** As Fig. 13, but with $\gamma = 0.55$ and $\sigma = 32$. All other parameters are equal to or set randomly as in Fig. 13.

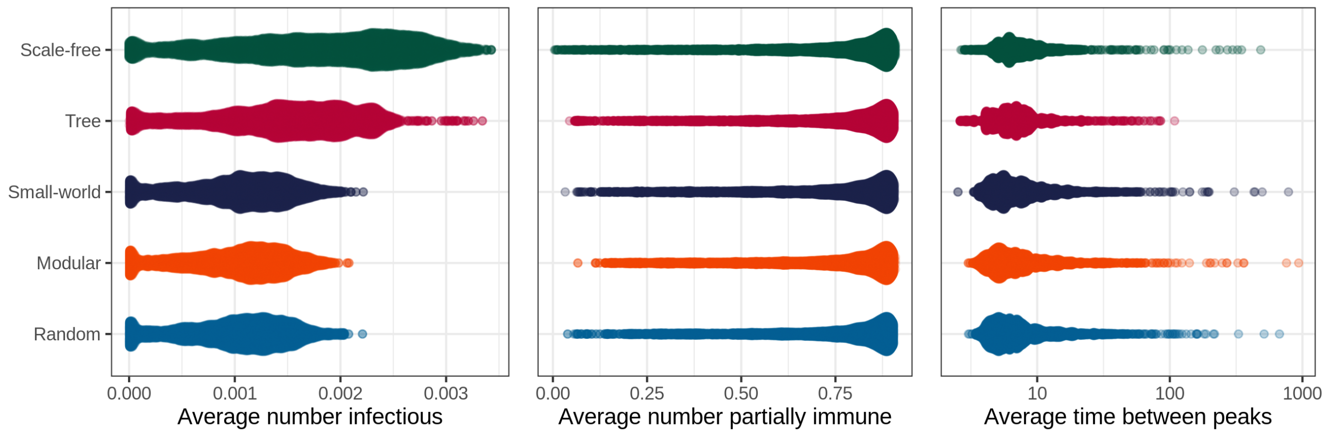


**Figure 18.** As Fig. 14, but with $\gamma = 0.55$ and $\sigma = 32$. All other parameters are equal to or set randomly as in Fig. 14. We note that the relative positioning of the networks' average cycle length differs from the parameters used for the simulations presented in the main text, with scale-free networks here having shorter cycles on average, compared to longer cycles on average in the main text parameterization.

## References


**1.** Gupta, S., Ferguson, N. & Anderson, R. Chaos persistence, and evolution of strain structure in antigenically diverse infectious agents. *Science* **280**, 912–915 (1998).

**2.** Lourenço, J. & Recker, M. Natural, persistent oscillations in a spatial multi-strain disease system with application to dengue. *PLOS Comput. Biol.* **9**, e1003308 (2013).

**3.** Lourenço, J., Wikramaratna, P. S. & Gupta, S. MANTIS: an R package that simulates multilocus models of pathogen evolution. *BMC Bioinforma.* **16** (2015).

**4.** Anderson, R. M., Jackson, H. C., May, R. M. & Smith, A. M. Population dynamics of fox rabies in europe. *Nature* **289**, 765–771 (1981).
